# The making of a genomic parasite - the *Mothra* family sheds light on the evolution of *Helitrons* in plants

**DOI:** 10.1186/s13100-015-0054-4

**Published:** 2015-12-17

**Authors:** Stefan Roffler, Fabrizio Menardo, Thomas Wicker

**Affiliations:** Institute of Plant Biology, University of Zürich, Zollikerstrasse 107, Zürich, CH-8008 Switzerland

**Keywords:** Transposon, Helitron, RPA, Rice, Horizontal transfer

## Abstract

**Background:**

Helitrons are Class II transposons which are highly abundant in almost all eukaryotes. However, most Helitrons lack protein coding sequence. These non-autonomous elements are thought to hijack recombinase/helicase (RepHel) and possibly further enzymes from related, autonomous elements. Interestingly, many plant Helitrons contain an additional gene encoding a single-strand binding protein homologous to Replication Factor A (RPA), a highly conserved, single-copy gene found in all eukaryotes.

**Results:**

Here, we describe the analysis of *DHH_Mothra*, a high-copy non-autonomous Helitron in the genome of rice (*Oryza sativa*). *Mothra* has a low GC-content and consists of two distinct blocs of tandem repeats. Based on homology between their termini, we identified a putative mother element which encodes an RPA-like protein but has no *RepHel* gene. Additionally, we found a putative autonomous sister-family with strong homology to the *Mothra* mother element in the RPA protein and terminal sequences, which we propose provides the RepHel domain for the *Mothra* family. Furthermore, we phylogenetically analyzed the evolutionary history of RPA-like proteins. Interestingly, plant Helitron RPAs (PHRPAs) are only found in monocotyledonous and dicotyledonous plants and they form a monophyletic group which branched off before the eukaryotic “core” RPAs.

**Conclusions:**

Our data show how erosion of autonomous Helitrons can lead to different “levels” of autonomy within Helitron families and can create highly successful subfamilies of non-autonomous elements. Most importantly, our phylogenetic analysis showed that the PHRPA gene was most likely acquired via horizontal gene transfer from an unknown eukaryotic donor at least 145–300 million years ago in the common ancestor of monocotyledonous and dicotyledonous plants. This might have led to the evolution of a separate branch of the Helitron superfamily in plants.

**Electronic supplementary material:**

The online version of this article (doi:10.1186/s13100-015-0054-4) contains supplementary material, which is available to authorized users.

## Background

Helitrons are a superfamily of transposable elements (TEs) in eukaryotes which was discovered only relatively recently in *Arabidopsis thaliana*, *Caenorhabditis elegans* and *Oryza sativa* [1]. They have since been found in many genomes of flowering plants [1, 2], mosses [3], fungi [4–6] but also many animals such as sea urchin [7], fish [8, 9] and bats [10]. A recent *in silico* analysis using the program *Helsearch* [2] estimates the number of Helitrons in rice and sorghum to approximately 7000 and 5000, respectively, covering several megabases of their hosts' genomes. The most extensively studied genome regarding Helitrons is the one of maize, where approximately 2000 intact Helitrons and more than 20,000 Helitron fragments were found. Based on high homology between individual elements they are thought to still be very active [11]. As for most DNA transposons, the majority of Helitron elements are non-autonomous and do not encode any proteins. These non-autonomous elements presumably depend for their transposition on enzymes encoded by “mother” or “master” elements elsewhere in the genome.

One reason why Helitrons remained undiscovered for a long time is their limited diagnostic features. They lack terminal inverted repeats (TIRs) and the only motifs common to all Helitrons are the dinucleotide TC at the 5' end as well as a CTRR motif at the 3' end. Additionally, almost all Helitrons have a G/C rich 15–20 bp hairpin motif approximately 10–12 bp upstream of the 3' end, which is thought to serve as a stop signal in the transposition process [1]. Finally, Helitrons have a strong preference to insert between the bases A and T or sometimes between two Ts [1].

The transposition mechanism of Helitrons and the involved proteins differ from those of the well described DDE transposases. Autonomous Helitrons encode a RepHel protein of 1000–3000 amino acids (aa) length, which is thought to initiate the replication. The RepHel constitutes a replication initiation domain (RCR/Rep) followed by a helicase enzyme (Hel) of approximately 400 aa [12]. Because of structural homology with the catalytic core of HUH endonucleases of a bacterial rolling-circle transposons [13], it was suggested that Helitrons use a rolling-circle mechanism involving a single-stranded DNA intermediate for transposition and replication [1, 12]. Li and Dooner [14], however, clearly showed excisions of Helitrons from 0.4 to 6 kb size in somatic Maize tissue. This challenges the current model and suggests an alternative mode of transposition involving excision and repair similar to TIR transposons. Indeed, it is possible that single stranded DNA transposition can result in the elimination of that copy from that locus when occurring during S phase of meiosis 1 [15].

Even though Helitrons are ascribed to the Class II (DNA) transposons, they remain unique due to their exclusive structural features and transposition mechanism and belong to a separate subclass within the DNA transposons [16]. However, rolling-circle transposition mechanisms have been described for gemini viruses [17], plasmids and some bacterial transposons [18]. Structural homology between their transposases suggests very ancient origin of Helitrons [1]*.*

In plants, some Helitrons have been reported to also encode a distant homolog of the Replication Protein A (RPA), a protein ubiquitous in eukaryotes [19, 20]. RPA has several single-strand DNA binding sites and is involved in processes such as DNA replication and repair. RPA homologs have also been identified in Helitrons from zebrafish and sea anemone [12] and in Helentrons (a sub-type of Helitrons) in *Drosophila melanogaster* [21].

At least in maize, Helitrons seem to acquire close by gene fragments very frequently. Several studies showed an ongoing gene movement, gene shuffling and transcriptional read-troughts, which is attributed to Helitron activity [22, 23]. In the maize line B73, approximately 11,000 such chimeric transcripts have been found to be expressed which represents almost one quarter of all genes [24]. Therefore, it is thought that Helitrons contributed substantially to the recent diversification observed in the maize genus. Moreover, frequent gene capturing mediated by Helitrons was also reported in the silk worm *Bombyx mori* [25] and in the bat *Myotis lucifugus* [26].

In this study we describe the analysis and origin of a high-copy Helitron family in rice, which we named *DHH_Mothra.* Non-autonomous *Mothra* elements are present in hundreds or even thousands of copies in multiple rice species, which merited an in-depth analysis of this TE family. We identified a putative mother element for the *Mothra* family that encodes an RPA homolog but no RepHel protein. We moreover identified a closely related Helitron family, which we propose to be the donor for the lacking RepHel enzyme of *Mothras.* According to our model, this introduces an additional level of autonomy. We furthermore investigated the evolutionary background of Helitron RPA acquisition in plants and suggest horizontal transfer most likely from a unicellular eukaryote into the common ancestor of mono- and dicotyledonous plants.

## Results

### *Mothra* is a high-copy non-autonomous Helitron

In a previous study [27] we compared the two closely related rice species *O. sativa*, the Asian rice, with its relative *O. glaberrima*, the African rice, and investigated presence/absence polymorphisms of Class II transposons of the TIR subclass. While scanning polymorphic TE sites, we repeatedly encountered a sequence which was obviously of repetitive nature but we were unable to classify it at that time. Now, we found that it was in fact a non-autonomous TE of the Helitron order which we called *Mothra*.

**Fig. 1:**
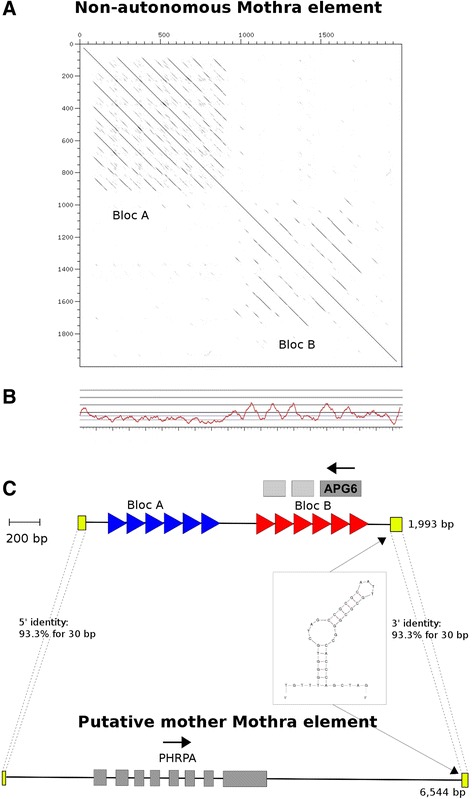
Overview of the non-autonomous *Mothra* consensus sequence and its putative mother element. **a** Dot-plot of the non-autonomous *Mothra* consensus sequence against itself shows the two repetitive Blocs A and B. **b** GC-plot of the non-autonomous element. Note that Bloc A shows a unusual low GC-content of approximately 20 %. **c** Schematic overview of the non-autonomous *Mothra* and its putative mother element below. Both elements share the characteristic hairpin structure at the 3' end. The termini of the putative mother element and the non-autonomous consensus are conserved (in yellow). Furthermore, the non-autonomous elements shows the putative ORF of 96 amino acids. Note here, that the putative mother element of *Mothras* encodes for a RPA homolog, which we named PHRPA, but no RepHel protein

We identified a total of 1,682 *Mothra* elements from which we manually deduced consensus sequences of 22 sub-types. The 22 *Mothra* sub-types share the same terminal and internal sequence motifs but vary in size between 1252 and 2741 bp (see Methods). The differences in size between the sub-types are due to differences in the order, length and/or orientation of blocs of tandem repeats (see below). From these 22 sub-types, we created a single consensus sequence of 1993 bp in length which we refer to as consensus of the non-autonomous *Mothra* elements (Fig. 1a). As described for other Helitrons, *Mothra* elements show the characteristic dinucleotide TC at its 5' end and the four bases CTAG at the 3' end. Additionally, we found the characteristic hairpin motif of 16 bp length located 13 bp upstream of the 3' end of the elements. From this, we concluded that *Mothra* is in fact is a non-autonomous TE of the Helitron order.

### *Mothra* contains tandem repeats and gene fragments

*Mothra* contains two distinct sequence blocs (Bloc A and B, Fig. 1a). Bloc A, which ranges approximately from position 80 to position 900 in the consensus sequence, consists of six direct repeats and shows a very low GC content of 20 %. Bloc B ranges from position 950 – 1860 and consists of six different, less conserved direct repeats and exhibits an average GC content of about 40 % (Fig. 1b). There is great variety in the number of the repeat units within the Blocs A and B among the individual copies. In some cases, the order of the blocs is even reversed. In other cases, additional sequence is present between or sometimes even within one of the two blocs.

By definition, non-autonomous elements do not encode any proteins. But interestingly, the *Mothra* consensus sequence contains a putative open reading frame (ORF) of 96 amino acids in reverse orientation in Bloc B. The predicted protein shows sequence homology to the APG6 domain (Pfam ID: pfam04111, e-value: 2,2e-03) which has been described to be involved in autophagy and vascular sorting pathways in yeast [28]. Because of the repeat structure of Bloc B, this homology is partially repeated two more times downstream of this ORF. These additional copies, however, lack start codons and therefore do not constitute intact ORFs. We assume that this ORF is the result of gene fragment capture but probably has no function. The fact that this gene fragment is part of the *Mothra* consensus sequence indicates that the gene capture event occurred before the radiation of the *Mothra* family.

### The putative *Mothra* mother element lacks a *RepHel* gene

**Fig. 2:**
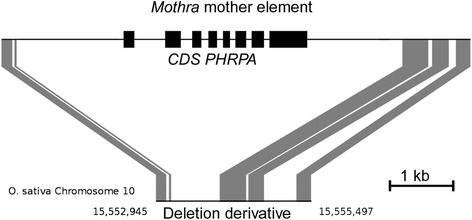
Schematic representation of an identified deletion derivative of the putative mother element of *Mothras*. The coding sequence for the *PHRPA* gene is indicated in black while the homologous sequences are indicated in gray

Usually, non-autonomous TEs share their terminal sequences with their autonomous “mother” elements. That is why we scanned the genome of *O. sativa* using the first 50 and the last 80 bp of the non-autonomous element, respectively, as queries. We extracted 323 sequences where we identified both ends in the same orientation located within 25 kb from each other. We scanned the 323 fragments for the presence of transposases and helicases but could not identify a single one. However, we identified one sequence of 6544 bp in length that encodes an RPA homolog (Fig. 1c). This RPA sequence was annotated in the rice genome as hypothetical protein (LOC_Os11g47400). The predicted protein contains several generic single-stranded DNA-binding sites. After manual re-annotation of the protein we were able to extend the putative protein length from 296 aa to 472 aa and the number of exons from four to eight. Interestingly, this sequence was the only one among all 323 analyzed fragments containing a putative complete gene between the two *Mothra* ends. The sequence homology between the termini of this putative mother element and the non-autonomous *Mothra* consensus is very high (93,3 % of the terminal 30 bp, and 81,2 % and 80,2 %, respectively for the terminal 100 bp). According to Yang et al. [2], this makes them not only members of the same family but also of the same sub-family. Moreover, we identified a deletion derivative of the putative mother element that shows homology to almost the entire element but lacks the RPA domain (Fig. 2). This indicates that we indeed identified a distinct element rather than an RPA homolog that is flanked by chance by two fragments of termini from non-autonomous *Mothra* elements. Therefore, we propose this element, even if we did not find an ORF encoding an RepHel protein, to be the mother element of the numerous non-autonomous *Mothras*. Thus, in the strict sense, the putative *Mothra* mother element might itself not be autonomous (see before).

### Polymorphisms between *O. sativa* and *O. glaberrima* demonstrate recent activity of *Mothra* elements

**Fig. 3:**
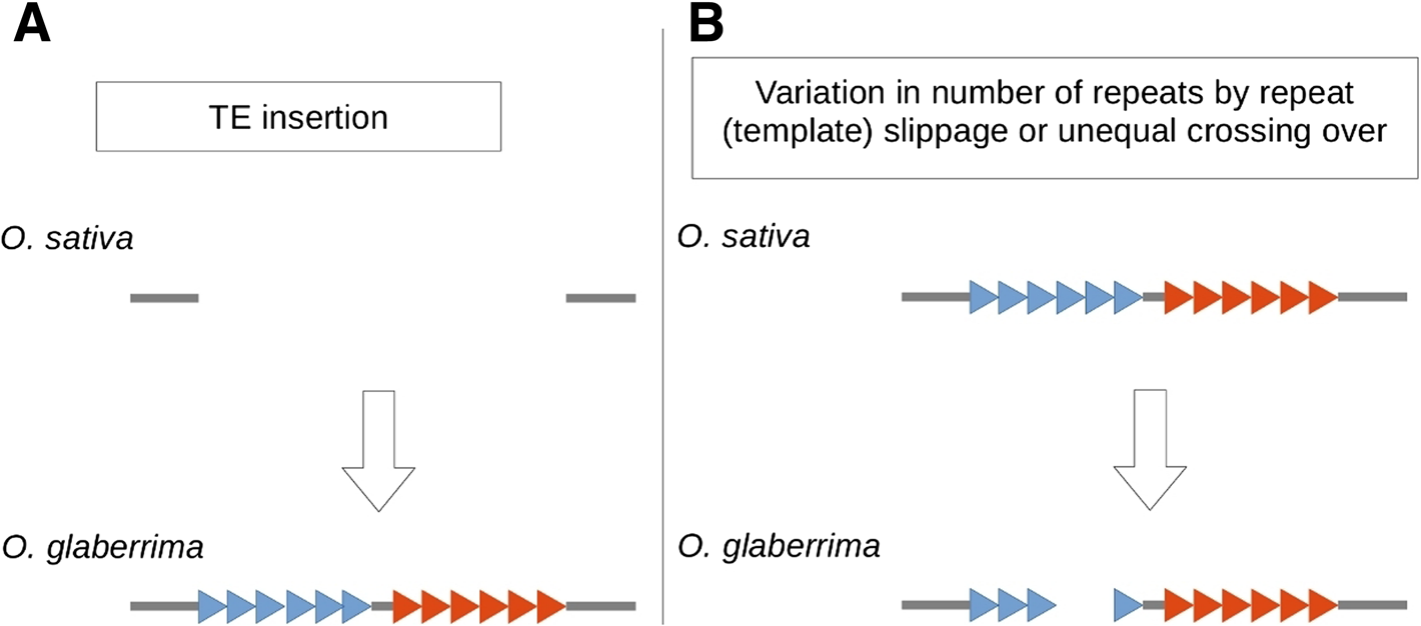
Presence/absence polymorphisms of *Mothra* elements between *O. sativa* and *O. glaberrima. O. sativa* and *O. glaberrima* diverged approximately 600.000 ya. **a** True polymorphisms are characterized by presence/absence of whole elements. We found a total of four insertions and four putative excisions. **b** Most of the variation in the two *Mothra* populations comes from repeat-slippage or unequal crossing-over

In a previous study we produced an alignment of approximately 60 % of the *O. sativa* and *O. glaberrima* genomes for identification of presence/absence polymorphisms of TIR transposons [27]. Now, we searched this alignment for polymorphisms related to *Mothra* elements. Out of a total of 856 *Mothra*-related polymorphisms, we investigated 148 manually. Most of them turned out not to be actual presence/absence polymorphisms, but rather variations in the number of repeat units between orthologous *Mothras* of the two species. Most of these differences probably arose from mechanisms such as unequal crossing-over or repeat slippage rather than from transposition activity (Fig. 3). Thus, the vast majority of *Mothra* copies are found in the same position in both rice species, meaning that they inserted before the two species diverged approximately 600,000 years ago [29]. Therefore, we can say that most of the copies are older than 600,000 years.

**Fig. 4:**
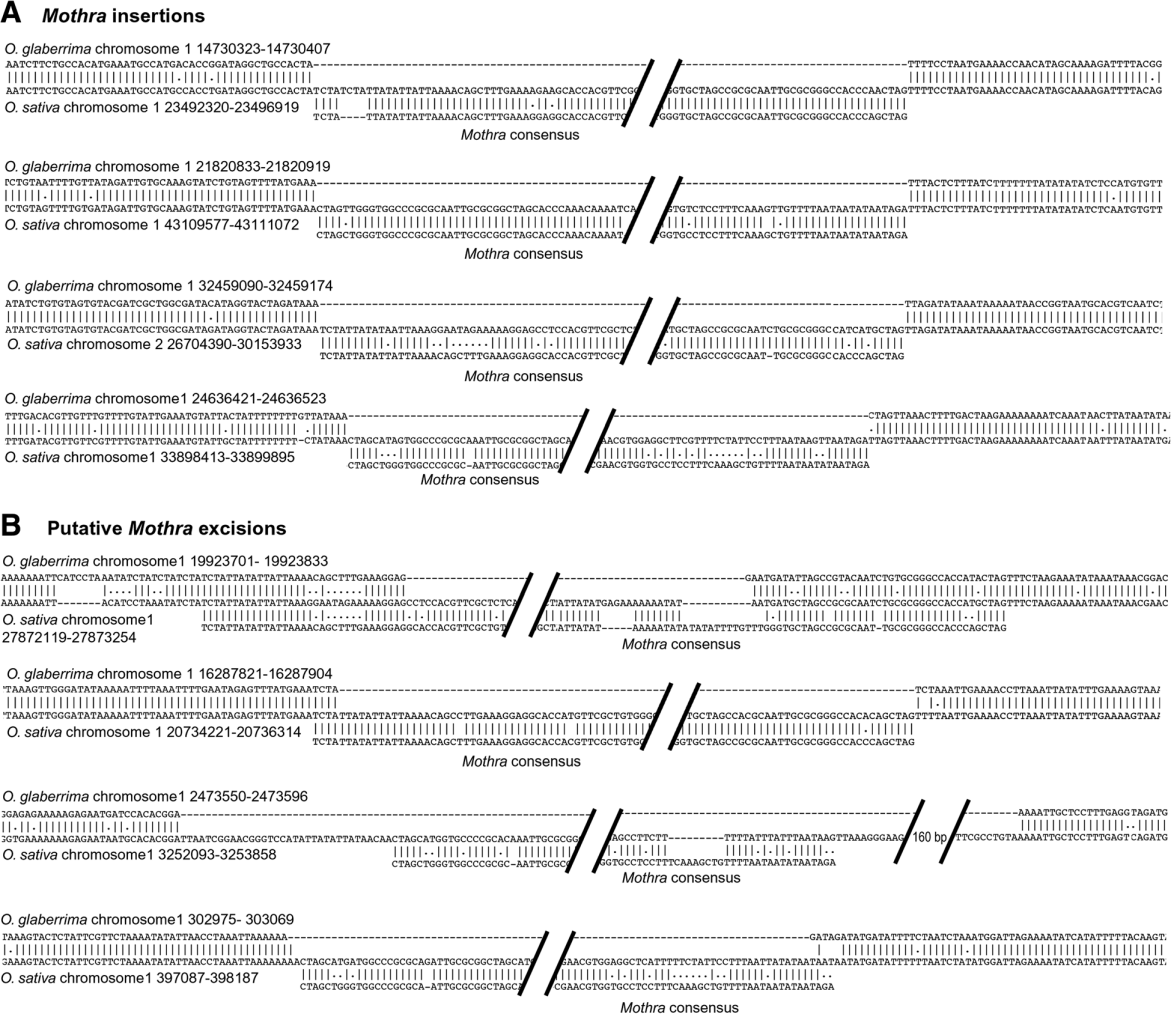
Examples of polymorphic *Mothra* elements in *O. sativa* and *O. glaberrima*. Shown are the alignments of the orthologous loci from *O. sativa* and *O. glaberrima*. The Mothra consensus sequence is aligned underneath. **a** *Mothra* insertions in *O. sativa*. The *Mothra* elements insert into the genome without producing a target site duplication. **b** Putative excision events in *O. glaberrima*. DNA repair patterns are similar to those found for DNA transposons. They include incomplete excision of the element (top two alignments), deletions in the flanking regions (third alignment) or insertion of filler sequences (bottom alignment)

However, we also identified eight sites where we found putative insertion/excision polymorphisms of non-autonomous *Mothras* between the two rice species (Fig. 4a). In four cases, we found the *Mothra* element located between the characteristic nucleotides A and T present in *O. sativa* but not in *O. glaberrima.* Because Helitrons do not generate target site duplications, these events probably represent typical insertions in *O. sativa.* Interestingly, we found four sites where we suspect putative *Mothra* excisions. We conclude this based on the DNA repair patterns which are similar to those described for TIR DNA transposon excisions [30] (Fig. 4b). In two cases, we observed incomplete excision events whereas the other two cases went along with a deletion and the introduction of filler DNA, respectively.

The eight polymorphic elements correspond to 5,4 % of subset of 148 manually investigated polymorphisms. Considering that we identified a total of 856 insertion/deletion polymorphisms between the two species, we extrapolate that a total of approximately 46 *Mothra* elements have moved since the two species diverged about 600,000 years ago [29]. However, this number is based on approximately 60 % of the genome which was aligned. Thus, the actual number of transposed elements might be even higher. Compared to the previously investigated TIR transposons [27], we conclude that *Mothra* has a level of activity similar to that of highly active DTT-Mariner elements.

### Phylogenetic analysis of the *Mothra* RPA homolog family

**Fig. 5:**
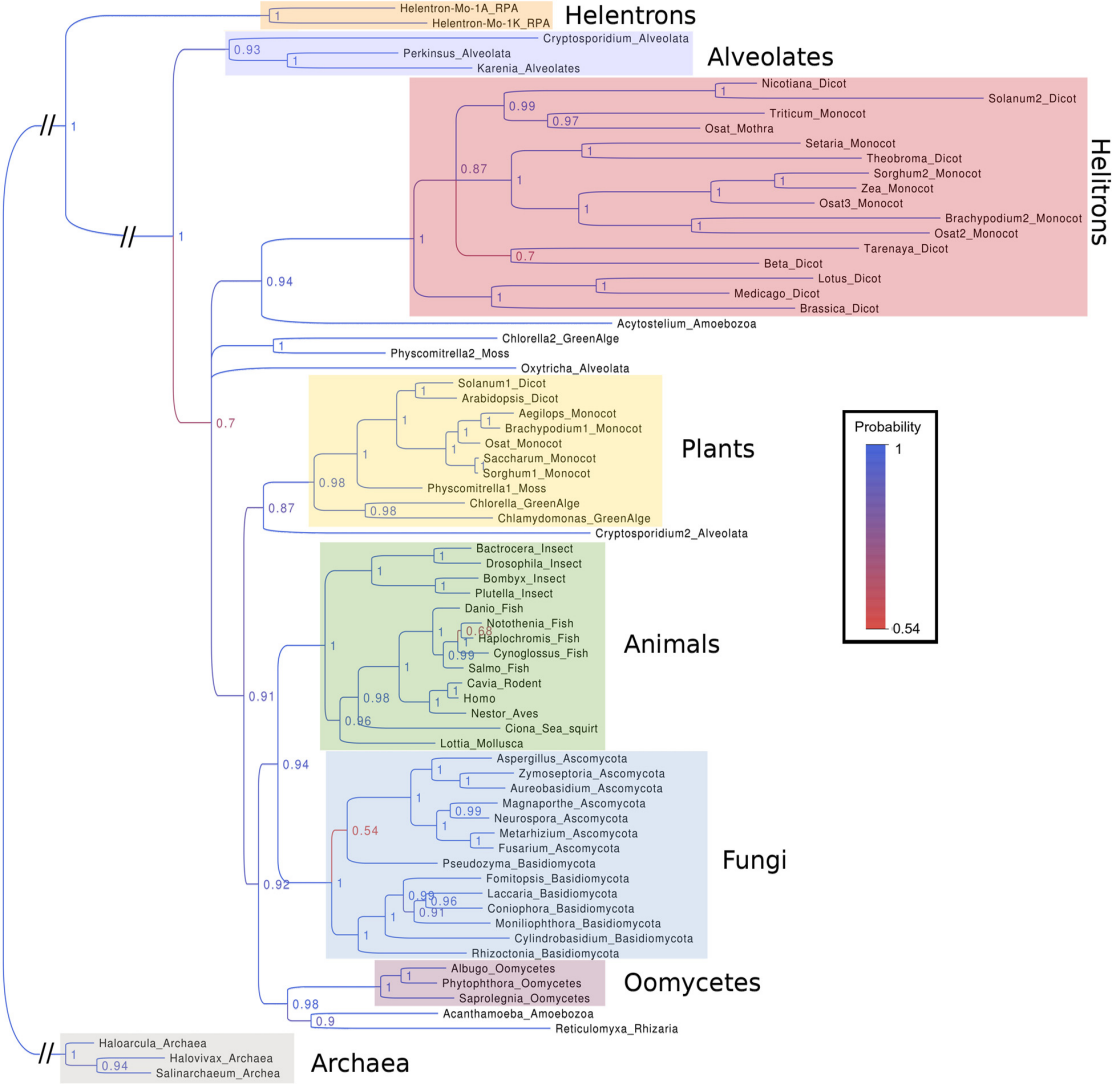
Phylogenetic tree of 72 RPA homologs from 62 species. We found clear monophyletic grouping of the original RPA protein into the “core” eukaryotic clades such as plants, animals, fungi, Oomycetes and Alveolates. Within the clades the taxonomy widely reflects the diversification within these clades. The Helitron clade, which includes the *Mothra* RPA protein, however, forms an independent branch. Interestingly, it includes only representatives of the monocotyledonous and dicotyledonous plants. For the phylogeny we used a maximum likelihood approach using the software MrBayes (reversible jumping Markov chain Monte Carlo (MCMC) simulations for 4 million generations). The numbers at branches reflect confidence values (i.e. probabilities that sequences to the right of the fork group together in 6000 generated trees)

RPA proteins are involved in crucial processes such as DNA-replication and -repair. Furthermore, this “core” RPA is a single copy gene and highly conserved among eukaryotes. This makes RPA useful for phylogenetic analysis and, thus, to study the origin of the plant Helitron RPA homolog (PHRPA). We used the the original “core” RPA as well as identified *Mothra* PHRPA of *O. sativa* as queries for NCBI blast searches against representatives from all major eukaryotic branches. We also included species from the largely under-sampled unicellular eukaryotic clades, such as Alveolata, Amoebae, Oomycetes and Rhizaria. Furthermore, we include two RPA homologs from Helentrons that were identified in *Drosophila melanogaster* [21] to investigate their relationship to PHRPAs. As an outgroup, we used some distant homologs from archaea (Fig. 5). Except in monocotyledonous and dicotyledonous plants, we usually found exactly one *RPA* gene (see below). The final dataset comprised 72 proteins from 62 species.

Our results show that most major eukaryotic clades cluster in monophyletic groups. We observe a clear grouping into plants, animals, fungi and Oomycetes. The phylogeny within these clades is consistent with the established taxonomy of eukaryotes [31]. For example plant RPAs first split into algae, mosses and later into monocots and dicots (Fig. 5). Because of the robustness of the tree and the great concordance with the taxonomy, these proteins most probably represent the intrinsic, eukaryotic “core” RPAs.

Most clades have exactly one RPA gene but there are exceptions. Interestingly, one of the two copies obtained from the Alveolata, *Cryptosporidium,* also clusters at the root of the plant branch. However, the other copy we find, as expected, in the clade of Alveolates, which are even more distant to the core RPA clade than the PHRPA family. Furthermore, we found two RPA paralogs in the genomes of *Physcomitrella*, a Moss, and the green alga *Chlorella*, to form a monophyletic group on the same level as the PHRPAs. It is possible that there are contaminations since these organisms are difficult to isolate and cultivate.

Most importantly, we find the PHRPAs to form a separate, monophyletic group outside the core RPA clade. Thus, we conclude that the PHRPA ancestor protein has evolved very early in the transition from prokaryotes to eukaryotes. Interestingly, we only find representatives of mono- and dicotyledonous plants in the PHRPA clade. Moreover, PHRPAs are more diverse than core RPAs. Indeed, PHRPA proteins are on average 21 % identical to each other, while core RPAs show an average of 39 % sequence identity (Additional file 1: Figure S1). Also the branch lengths of the PHRPA clade are noticeable long. This suggests diversification of new, independent gene subfamilies. The possible reasons why these proteins are only found in monocots and dicots which diverged approximately 145–300 million years ago (mya) [32, 33] are discussed below (see discussion). Moreover, our analysis reveals that the RPA proteins acquired by Helentrons seem to be of another, even more distant origin. These proteins form a separate group which branches off before the radiation of eukaryotes (Fig. 5).

### *Mothras* might use the RepHel protein of closely related Helitrons

Above, we describe that the putative mother element of the non-autonomous *Mothras* encoded an PHRPA protein but not for a RepHel protein. This raises the question of how these elements would actually transpose. As it has been described for non-autonomous elements, that they recruit closely related transposases, we suspect that RepHel from a closely related Helitron family would be used by *Mothra* elements. Therefore, we scanned the *O. sativa* genome for homologs of the PHRPA protein and extracted 21 fragments including 20 kb up- and downstream of the protein. Out of these we identified nine sequences with sizes from 8064 to 15,513 bp that all contain a *PHRPA* homolog and an adjacent *RepHel* gene.

**Fig. 6:**
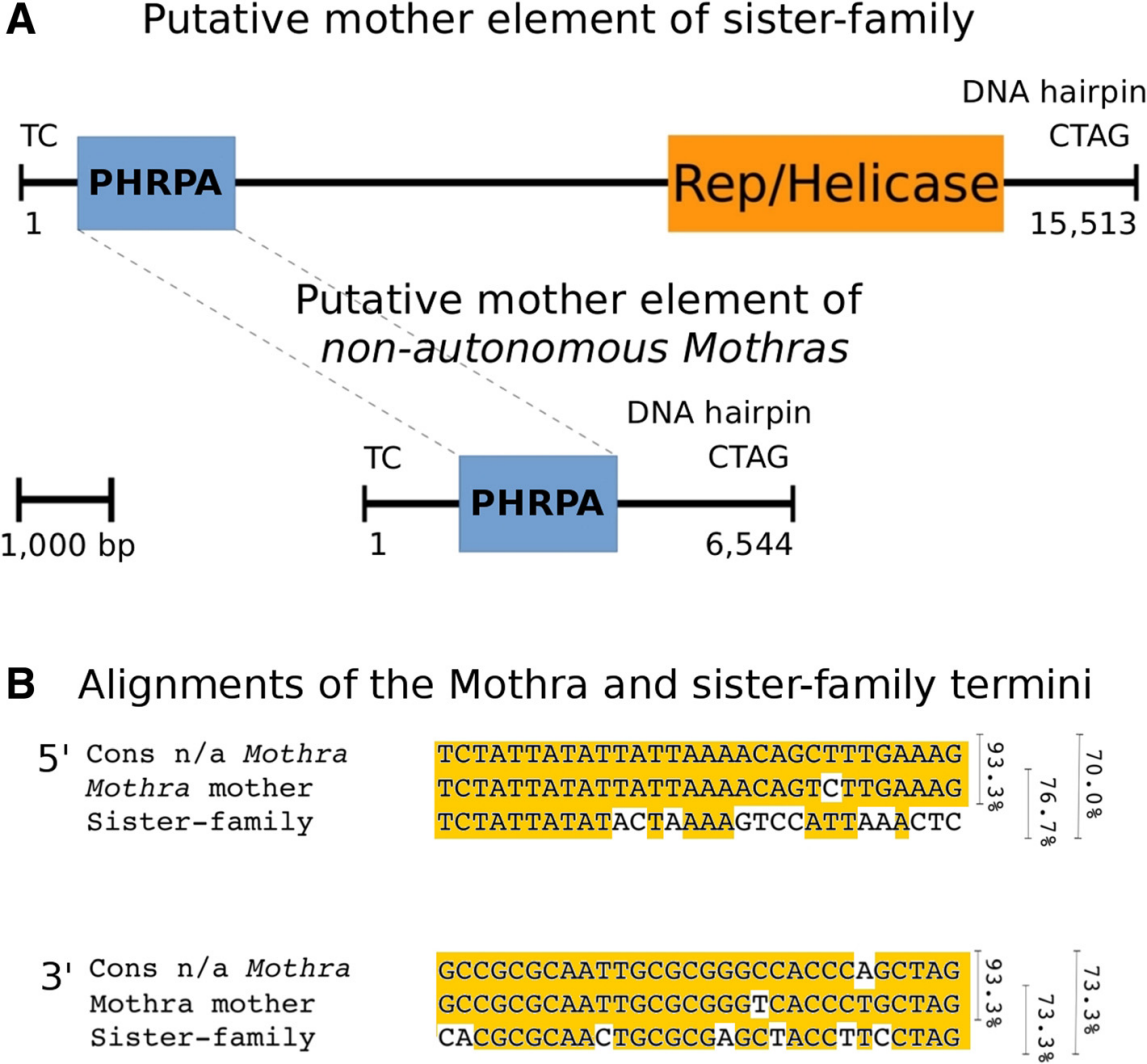
Schematic overview of a putative autonomous sister element of *Mothra.* **a** The element encodes a RPA protein closely related with the *Mothra* RPA (PHRPA) and, most importantly, also a RepHel protein. **b** The alignment shows the terminal sequences of the non-autonomous *Mothra* consensus, the putative mother element and the *Mothra* sister-family

Based on sequence homology we could clearly differentiate them into three groups. While we found five copies of group 1 elements, there were two copies each for groups 2 and 3, respectively. The PHRPA of group 1 is most similar to that of the Mothra mother element (46.1 % similarity compared to 21.6 % and 22.6 % for groups 2 and 3, respectively). Moreover, the elements of group 1 and *Mothras* nearly fulfill the criteria of Yang et al. [2] to belong to the same family (73 % identity over 30 bp at the 5' end and 77 % identity at the 3' end). Because of this and the strong homology of their RPA proteins, we henceforth refer to these Helitrons of as the sister-family of *Mothra* (Fig. 6). Interestingly, when we compared the five copies of the sister-family with those in *O. glaberrima*, we found all of them to be polymorphic (Table 1), indicating recent activity of the *Mothra* sister-family. Thus, we propose that *Mothra* elements recruit the RepHel protein of their sister-family to transpose. For both, the *PHRPA* gene of the *Mothra* mother element and *PHRPA* and *RepHel* of the sister-family, we found transcripts in NCBI, suggesting that both might still be active (Additional file 2: Table S1).

**Table 1:** Overview of all identified copies of the putatively autonomous elements of the *Mothra* sister-family in *O. sativa* and *O. glaberrima*

*Mothra* sister-family copies			
*O. sativa*			
	Start pos.	End pos.	Comment
Chromosome			
11	26,634,911	26,619,399	Reverse
11	22,184,151	22,168,642	Reverse
11	24,183,680	24,199,188	Forward
5	592,132	606,965	Forward
5	25,964,570	25,949,203	Reverse
*O. glaberrima*			
	Start pos.	End pos.	Comment
Chromosome			
11	19,460,159	19,467,758	No RPA

## Discussion

The goal of our study was to characterize the origin and evolution of the high-copy Helitron family *Mothra* in rice. Although Helitrons are found in nearly all eukaryotic genomes they are much less well understood than other TE superfamilies. Despite their considerable role in exon shuffling and gene movement in plants [22–24], only few studies are available that shed light on their transposition mechanism. Initially, it was proposed that Helitrons replicate via a rolling-circle mechanism [1]. However, this was challenged by the discovery of Helitron excisions in somatic maize tissue [20]. Our data also suggest that some of the presence/absence polymorphism in rice might represent Helitron excisions. While Li and Dooner [14] mainly found repair patterns introducing TA micro-satellites as “filler” DNA, our putative excision events were also associated with deletions of the flanking sequences. These footprints strongly resemble those of TIR transposon excisions [27, 29, 34, 35]. Thus, these combined findings suggest the existence of at least one alternative transposition pathway to the proposed rolling-circle mechanism.

Despite these open questions, the main findings of our study provided insight into the evolution of different levels of non-autonomous elements and, more importantly, of the Helitron superfamily in plants in general. Our main conclusions are discussed in the following.

### Sequence composition of non-autonomous *Mothras* elements might play a role in transposition efficiency

Non-autonomous transposons can create hundreds or even thousands of copies in only few generations [36]. Loss of protein coding sequences and thereby autonomy has happened in all major Class II TE superfamilies. It can be explained by the fact that hosts regulate TEs via epigenetic silencing. Thus, constant reshaping, shortening and the accumulation of “nonsense” sequences might be mechanisms to avoid RNA silencing [37]. Alternatively, the presence of an active functional copy might release selection pressure on other copies, allowing for non-autonomous derivatives to emerge. Still, non-autonomous elements retain the ability to cross-mobilize related transposases. This type of trans-acting system has best been described in detail for the TIR transposons of the *DTT-Mariner* superfamily [36]*.* Transient expression experiments in yeast showed that the affinity for the autonomous element was determined by the TIR region. The efficiency of transposition, however, was influenced dramatically, positively or negatively, by different compositions of internal sequences.

We suspect that the great success of *Mothra* elements might have to do with their unusual sequence composition (see Fig. 1). The Blocs A and B of the non-autonomous element are unique to *Mothra* elements and their high conservation within the *Mothra* family suggests functional importance. When we screened the genomes of *O. sativa* and *O. glaberrima* for *Mothra* related polymorphisms (see above), we found that the majority of the differences were variations in the number of repeat units. Most likely these were caused by repeat slippage or unequal crossing-over for which the repeat arrays of Blocs A and B served as templates. Thus, these repeat arrays may be a sources of plasticity and permanent turnover within non-autonomous *Mothra* elements.

### The *Mothra* RPA homolog likely originated from horizontal transfer

In our phylogenetic analysis of RPA proteins we found clear monophyletic clustering of the “core” RPAs in all major eukaryotic groups which broadly reflects the separation of early eukaryotes into distinct lineages (see Fig. 5). Interestingly, the clade representing the RPA homologs from plant Helitrons (PHRPAs) branches off even before the separation of plants, animals, fungi and Oomycetes, indicating a very ancient origin of these proteins. It is the more surprising that this clade only includes proteins from monocotyledonous and dicotyledonous plants which only separated approximately 145–300 mya [32, 33]. Previous studies proposed that plant Helitrons hijacked and modified the eukaryotic core *RPA* gene which later became the plant Helitron RPA [1, 38]. However, the clear monophyletic origin of PHRPAs outside the core RPA clade challenges this model.

**Fig. 7:**
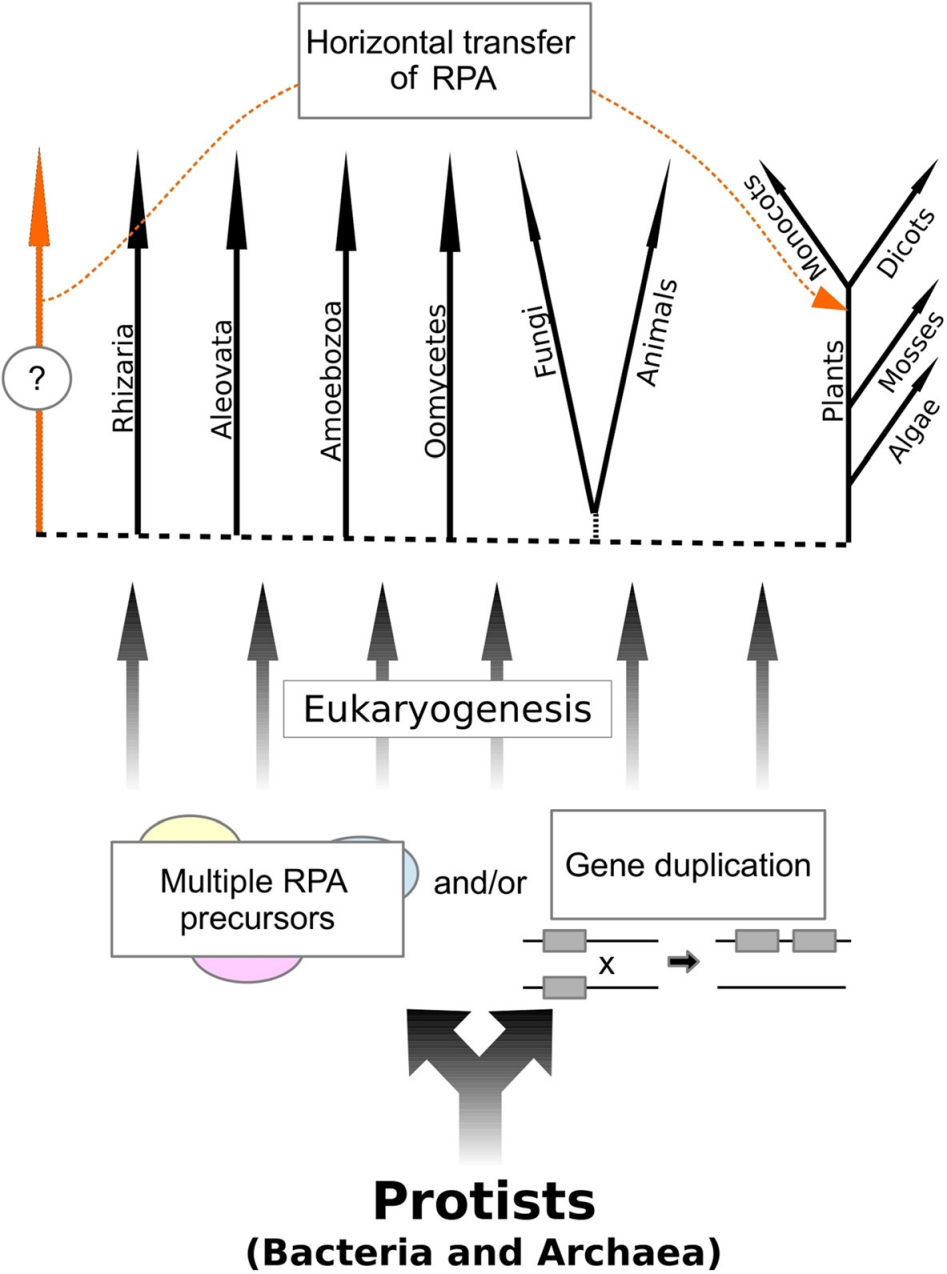
Schematic model of the horizontal transfer of PHRPA from an unknown donor. We found multiple RPA homologs in some species. It remains unclear if this is the result of gene duplication events in these lines and/or if there were multiple RPA precursors from different donors. Phylogenetic analysis indicates that PHRPA has evolved in parallel to the eukaryotic core RPA protein and was later introduced into the common ancestor of mono- and dicotyledonous plants. The PHRPA donor, however, remains unknown

There are two possible explanations for the phylogenetic position of PHRPAs: First, PHRPA proteins were originally present in all other eukaryotes and were lost in all lineages except the monocots and dicots. We consider this highly unlikely. The second explanation (which we clearly favor) is horizontal gene transfer. Typical characteristics of horizontal gene transfer are phylogenetic incongruence and/or unusually high sequence identity of proteins from otherwise distantly related species. In our case, we found very well supported phylogenetic incongruence. However, we could not identify a putative donor of PHRPA. This donor was obviously not sampled in our collection. We propose that PHRPA was transfered from this unknown and distantly related eukaryote into the progenitor of monocots and dicots. This horizontal transfer must have occurred before monocots and dicots diverged 145–300 mya [32, 33], since we have not found PHRPAs in any other plant group that diverged earlier*.* Our data indicate that the progenitor of all eukaryotic RPA genes was already present during eukaryogenesis, but it remains unclear if the last eukaryotic common ancestor had one or several RPA homologs (Fig. 7), because in several organisms such as *Physcomitrella*, *Chlorella, Acanthamoeba* and *Cryptosporidium* we find both, a core RPA and a homolog that is equally distant from the core RPA as the PHRPA clade. We therefore suspect that the donor of plant Helitron RPA homologs was probably a basal eukaryote similar to those mentioned above.

In Prokaryotes (bacteria and archaea), horizontal gene transfer is common and and it is believed to be a major mechanism for adaptation [39]. It becomes more and more evident that horizontal transfer is also a common process in eukaryotes. For example the extremophilic red alga *Galdieria sulphuraria* exhibits a enormous metabolic flexibility which it acquired by various genes from different bacteria and archaea [40]. Like genes, also TEs (if they are not the vector for gene transfer themselves) can be transfered between hosts. Often this involves intermediate vectors such as blood feeding insects or pathogens carrying bacteria or viruses to their new hosts. For example in 24 species of the insect order Lepidoptera two non-autonomous *Helitrons* were identified which were also found in the genomes of several double-stranded DNA polydnaviruses [41]. In plants, up to two million horizontal TE transfers only of LTR-retrotransposons were suggested by a comparative analysis among flowering plants [42].

However, what makes the case of PHRPA special is that the proposed horizontal transfer resulted in a successful new type of TE whose widespread distribution in monocots and dicots suggests advantages over normal Helitrons lacking this gene. Indeed, Dong et al. [43] described how stepwise acquisition of gene fragments can produce elements of increasing complexity.

Interestingly, our analysis also suggests that RPA homologs in *Drosophila*, called Helentrons, might also have been acquired though horizontal transfer. But the phylogenetic analysis indicates that they are of an even more distant origin. Furthermore, highly divergent RPA homologs were also found in Helitrons of zebrafish and starlet sea anemone [12]. However, here we were not able to identify any homology to PHRPAs, which is why they were not included in our phylogenetic analysis. Thus, it appears that Helitrons acquired single-strand binding proteins at least three times independently during evolution, suggesting convergent evolution.

### A model for the evolution of semi-autonomous and non-autonomous plant Helitrons

Our data suggest that the numerous non-autonomous *Mothra* elements are mobilized by a single mother element. Surprisingly, this putative mother element encodes for PHRPA but not for a RepHel protein. We speculate that the mother element might itself be depending on a related and fully autonomous element. Indeed, we found one candidate Helitron family that shows strong homology with the RPA protein and the termini of the *Mothra* mother element. We referred to that Helitron family as the *Mothra* sister-family.

**Fig. 8:**
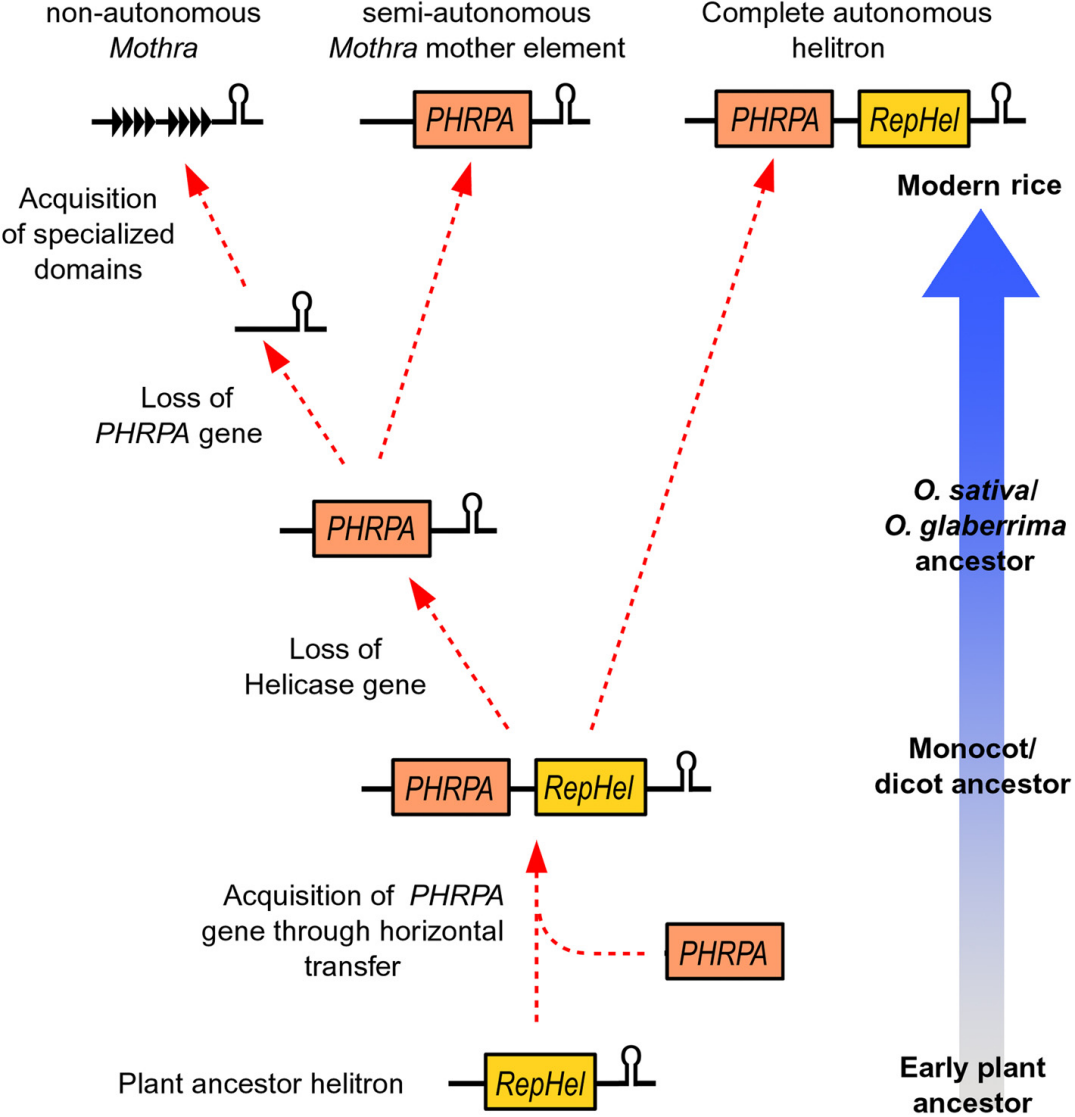
Schematic model of the evolution of plant Helitrons. We propose that the progenitor of plant Helitrons contained only a RepHel domain. Later, the PHRPA protein was introduced into the progenitor of mono- and dicotyledonous plants approximately 145 – 300 mya via horizontal transfer to form the first plant RPA-like Helitron. In the case of *Mothra* elements, the RepHel domain was later lost, thus introducing an additional level of “semi-autonomy” between the non-autonomous elements and the fully autonomous Helitrons

Based on these observations, we propose a model which introduces the putative mother element as an additional level of “semi-autonomy” (Fig. 8). We assume that the ancient Helitron consisted of a *RepHel* gene and probably the structural features like the 3' hairpin that we find to be common in all Helitrons. According to our model, the PHRPA protein was then introduced in the common ancestor of mono- and dicots via horizontal transfer 145–300 mya [32, 33] where it got acquired by the progenitor of all RPA containing plant Helitrons (discussed above). We propose that at a later point, one Helitron lineage lost its *RepHel* gene, resulting in the putative *Mothra* mother element that only contains the *PHRPA* gene. This semi-autonomous element would still fulfill some functions in the transposition process but would rely on the RepHel protein provided by the *Mothra* sister-family. Loss of internal sequences is common during transposition of Helitrons [43]. Furthermore, the evolution of non-autonomous transposable elements has been described in virtually all TE superfamilies [16].

According to our model, the next step in *Mothra* evolution was the loss of the *PHRPA* gene, resulting in a completely non-autonomous element that relies both on the *Mothra* mother element and functional copies of the *Mothra* sister-family (Fig. 8). Finally, the non-autonomous Mothra element acquired the complex tandem repeat blocs which, we propose, improved its transposition efficiency. This proposed stepwise evolution ultimately led to the situation we find in modern rice species where all three types of elements (fully autonomous, semi-autonomous and non-autonomous) exist side-by-side. However, biochemical assays will be needed to confirm the functional relationship between the described elements.

## Conclusion

Analysis of the *Mothra* family of Helitrons has provided unexpected insight in to the early evolution of plant Helitrons through the identification of a putative horizontal gene transfer that resulted in a successful sub-group of the Helitron superfamily. Furthermore, the great success of the non-autonomous *Mothra* elements suggests that combinations of different levels of transposition autonomy might be particularly efficient in Helitrons.

## Methods

### *Mothra* annotation

To generate the *Mothra* consensus sequence, we extracted and aligned 100 putative copies including 5 kb of flanking sequence which we used to manually determine the boundaries of the element. The identified termini matched the previously described canonical Helitron termini [16]. To deduce the consensus sequences for the sub-types and finally the consensus sequence of the non-autonomous *Mothra* element, we used the multiple alignment software Clustal X [44], the graphical dot-matrix program Dotter from the SeqTools package (https://www.sanger.ac.uk/resources/software/seqtools/) and in-house Perl scripts which are available upon request. To annotate *Mothra* elements we used the *Mothra* consensus sequence in Blastn searches against the *O. sativa Nipponbare* cultivar genome (Version 5) provided by the International Rice Genome Sequencing Project (IRGSP) (plantbiology.msu.edu/pub/data/) [45]. We included hits with a minimum length of 80 basepairs and at least 80 % identity. Because we found many fragments, we merged all hits that were found within 200 bps of flanking sequence to single hits.

To identify the *Mothra* mother element we used Blastn searches of the first 50 and the last 80 bps of the *Mothra* consensus sequence. We considered fragments where we found both ends in the same orientation and that were located within 25 kb from each other. We used the online NCBI platform (http://blast.ncbi.nlm.nih.gov/Blast.cgi) to perform Blastn and Blastx searches against the 323 putative sequences to identify the *RPA* gene. To identify the polymorphisms between *O. sativa* and *O. glaberrima* we used the whole genome alignment produced in a previous study [27].

### Phylogenetic tree

The sequences for the phylogenetic tree were retrieved from the NCBI database (http://www.ncbi.nlm.nih.gov/). We used the sequences of the identified Mothra RPA and the core RPA of *O. sativa* as queries and searched each of the main eukaryotic groups, animal, fungi, plants, Alveolata, Amoebae, Rhizaria, Oomycetes and archaea separately. We aligned them using Clustal X [44] with the following parameters for multiple alignments: Gap opening penalty of 10 and Gap extension penalty of 0.1. The phylogenetic tree was generated using MrBayes 3.2.2 [46]. We conducted two runs with 4 chains, each for 4 million generations, sampling every 500 generations. We used all the protein models available in MrBayes and used a reversible jump Monte Carlo Markov Chain (MCMC) [47]. Heterogeneity of substitution rates among different sites was modeled with a gamma distribution. The first quartile of generations was discarded (burn-in) and convergence was evaluated with the average standard deviation of split frequencies (0.002). To illustrate and re-root the tree we used the program Figtree (http://tree.bio.ed.ac.uk/software/figtree/).

### Data access

Sequences of *Mothra* elements were deposited in the TREP database (http://www.botinst.uzh.ch/research/genetics/thomasWicker/TREP.html). Sequence alignments that were used for phylogenetic analyses as well as in-house Perl scripts are available upon request.

## Additional files

Additional file 1: Figure S1. Distribution of identities between plant helitron RPAs and eukaryotic “core” RPAs. (PDF 274 kb) Additional file 2: Table S1. Transcripts that were identified, encoding the *Mothra PHRPA* gene and its sister-family *PHRPA* and *RepHel* genes, respectively. (PDF 42 kb)

## Abbreviations

TE: Transposable element
TIR: Terminal inverted repeat
aa: Amino acid
RPA: Replication protein A
ORF: Open reading frame
PHRPA: Plant helitron replication protein A
mya: Million years ago
