# Supplementary material for: The making of a genomic parasite - the Mothra family sheds light on the evolution of Helitrons in plants
Source: Mob DNA. 2015 Dec 17;6:23. doi: 10.1186/s13100-015-0054-4 (PMC4683698; doi:10.1186/s13100-015-0054-4)
Supplement: Additional file 1: Figure S1. — Distribution of identities between plant helitron RPAs and eukaryotic “core” RPAs. (PDF 274 kb) [file 13100_2015_54_MOESM1_ESM.pdf]

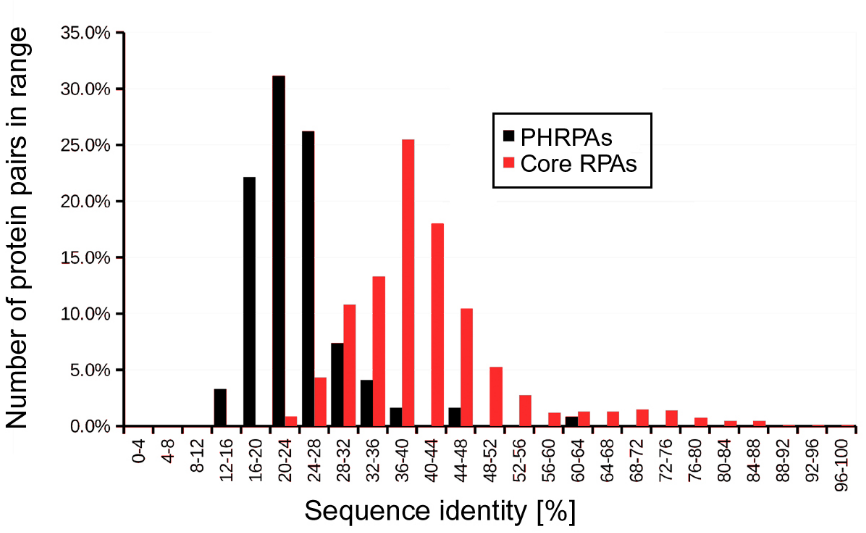

Additional Figure 1. Levels of sequence identity of RPA core and PHRPA proteins. For this analysis, all proteins within the two groups were compared pairwise. The x-axis shows the degree of sequence identity at the protein level while the y-axis shows the percentage of protein pairs in each class.
