# Supplementary material for: The making of a genomic parasite - the Mothra family sheds light on the evolution of Helitrons in plants
Source: Mob DNA. 2015 Dec 17;6:23. doi: 10.1186/s13100-015-0054-4 (PMC4683698; doi:10.1186/s13100-015-0054-4)
Supplement: Additional file 2: Table S1. — Transcripts that were identified, encoding the Mothra PHRPA gene and its sister-family PHRPA and RepHel genes, respectively. (PDF 42 kb) [file 13100_2015_54_MOESM2_ESM.pdf]

Additional Table 1. Transcripts of genes from the Mothra mother element and its sister-family.

Gene	GenBank accession
Mothra PHRPA	HS381803
Sister-family *PHRPA*	NM_001187565
Sister-family *RepHel*	CF306916
Sister-family *RepHel*	CF305553
